# Characteristics of Children in Foster Care, Family-Style Group Care, and Residential Care: A Scoping Review

**DOI:** 10.1007/s10826-016-0418-5

**Published:** 2016-04-04

**Authors:** Harmke Leloux-Opmeer, Chris Kuiper, Hanna Swaab, Evert Scholte

**Affiliations:** Horizon Youth Care and Special Education, Mozartlaan 150, 3055 KM Rotterdam, The Netherlands; Department of Clinical Child and Adolescent Studies, Faculty of Social and Behavioural Sciences and Leiden Institute of Brain and Cognition, Leiden University, Leiden, The Netherlands; Department of Clinical Child and Adolescent Studies, Faculty of Social and Behavioural Sciences, Leiden University, Leiden, The Netherlands

**Keywords:** Out-of-home care, Characteristics, Foster care, Family-style group care, Residential care

## Abstract

When risky child and family circumstances cannot be resolved at home, (temporary) 24-h out-of-home placement of the child may be an alternative strategy. To identify specific placement risks and needs, care professionals must have information about the child and his or her family, care history, and social-cultural characteristics at admission to out-of-home care. However, to date information on case characteristics and particular their similarities and differences across the three main types of out-of-home settings (namely foster care, family-style group care, and residential care) is largely lacking. This review compiles and compares characteristics of school-aged children of average intelligence and their families at the time of each child’s admission to one of the three care modalities. A scoping review technique that provides a broad search strategy and ensures sufficient coverage of the available literature is used. Based on the 36 studies included, there is consensus that the majority of normally intelligent children in care demonstrate severe developmental and behavioral problems. However, the severeness as well as the kinds of defining characteristics present differ among the children in foster care, family-style group care, and residential care. The review also identifies several existing knowledge gaps regarding relevant risk factors. Future research is recommended to fill these gaps and determine the developmental pathway in relation to children’s risks and needs at admission. This will contribute to the development of an evidence-based risks and needs assessment tool that will enable care professionals to make informed referrals to a specific type of out-of-home care when such a placement is required.

## Introduction

The United Nations Convention on the Rights of the Child states that every child has the right to live with his or her parents or to stay in touch with them, unless this would harm the child’s development (United Nations 1989). It also states that every child has the right to grow up in a supportive, protective, and caring environment that promotes his or her full potential. Positive child development is sometimes compromised by development-threatening child characteristics, adverse family circumstances, or interactions between both areas. When these risky circumstances cannot be effectively addressed by appropriate outpatient support, 24-h out-of-home placement of the child is usually considered a meaningful strategy for remediating the developmental risks (Bhatti-Sinclair and Sutcliffe 2012; Huefner et al. 2010; Pinto and Maia 2013; Vanschoonlandt et al. 2013).

Out-of-home (24-h) care consists of a continuum of intensive and restrictive care services, which range from lower-level family-based settings (e.g. relative foster care) to family-style group care to several types of residential treatment care (Huefner et al. 2010). Residential treatment centers in turn also reflect a continuum of services that vary from open residential to secure residential to inpatient psychiatric care (Barth 2002). Secure residential care seems to be especially preferred in juveniles with persistent aggressive behavior problems (Vermaes and Nijhof 2014), whereas inpatient psychiatric care is reserved for children who additionally display psychotic or suicidal behavior (Curtis et al. 2001; Huefner et al. 2010). In family-style group care, children live in home-like settings with live-in workers (Lee and Thompson 2009). This kind of care can be viewed as an intermediate setting between foster and residential care (Barth 2002; Huefner et al. 2010; Rouvoet 2009).

In accordance with the United Nations Guidelines for the Alternative Care of Children (henceforth “UN guidelines”), foster care or other family-based settings are the predominant types of care when out-of-home placement is required (United Nations 2009, December 18). These settings are considered to be most consistent with the best interests and needs of the child (Courtney 1998; Doran and Berliner 2001; Harder et al. 2013). However, little scientific evidence is available to support the recommendation to place children in family-based settings such as foster care (Bartelink 2013; Grietens 2012; Hussey and Guo 2002). In addition, one-third to one-half of foster children experience serious placement disruptions (Scholte, 1997; Van den Bergh and Weterings 2010; Van Manen 2011). These placement disruptions have negative impacts on children’s well-being and functioning. They also increase the risk of behavioral and emotional problems and heighten the likelihood of new (placement) breakdowns in subsequent foster families (Doran and Berliner 2001; Newton et al. 2000; Oosterman et al. 2007; Strijker et al. 2008). One of the main reasons for breakdowns in foster care is the child’s level of externalizing behavior problems (Barber and Delfabbro 2002; Newton et al. 2000; Strijker et al. 2008; Vanschoonlandt et al. 2012). Several researchers have therefore suggested that children with certain specific (treatment) needs are better off when they are placed directly in a more restricted treatment setting such as residential care (Barber et al. 2001; Butler and McPherson 2007; De Swart et al. 2012; Doran and Berliner 2001; Hussey and Guo 2002; Scholte 1997). Similarly, the UN guidelines state that residential care is applicable “for cases where such a setting is specifically appropriate, necessary and constructive for the individual child concerned and in his/her best interests” (United Nations 2009, December 18, p. 5). This statement implies that individual and contextual characteristics at the time of admission will partly determine which setting across the continuum of out-of-home care services is most appropriate. However, information on similarities and differences in a child’s attending risk factors and needs at the time of admission to a certain type of out-of-home care is to date largely unavailable or ambiguous (Barth 2002).

This paper compiles and compares child, family, care history, and social-cultural characteristics at admission of children who are placed in three of the main types of out-of-home care (namely foster care, family-style group care, and residential care). A scoping review technique is used to (1) chart case characteristics of normally intelligent children (aged 6–12 years) placed out-of-home in one of the three main care modalities, (2) define similarities and differences among those characteristics, (3) determine the severity of the child and family’s problems, and (4) identify the existing knowledge gaps within research on this particular population. The results of this scoping review will help practitioners and policy makers to be aware of specific risk factors and needs associated with children placed out-of-home, which might promote positive child development and reduce the risk of placement breakdowns. In addition, knowledge of these factors may contribute to the increased demand for an evidence-based assessment tool to determine these specific risks and needs of disturbed children; such as the Risk-Need-Responsivity model of Andrews et al. (2011).

## Method

We considered a scoping review to be the most fitting technique for answering our research question. Such a review provides a broad search strategy that includes hand searching through key journals, reference lists from the literature, and information from relevant organizations or existing networks (Arksey and O’Malley 2005). This technique is generally used to summarize research findings and identify research gaps (Arksey and O’Malley 2005). Hereto we used an adaptation of the developmental framework of Kerig et al. (2012). The framework of Kerig et al. (2012) is based on a holistic and dynamic approach that perceives a child’s development as being the result of interaction between a series of successive developmental processes. Simultaneously, the child interacts with his or her different contexts of development and deals with the attending risk and protective factors (Kerig et al. 2012). In line with this framework, we distinguished five contexts of development: (a) biological, (b) individual, (c) family, (d) care history, and (e) social-cultural.

The following inclusion criteria were used. Studies had to (a) focus primarily on child and family-related characteristics at admission that connect to the chosen developmental framework; (b) concern Western-oriented literature; (c) be written in English or Dutch; (d) have a publication date from 1990 onwards; (e) relate mainly to school-aged (i.e. 6–12 years) children; and (f) focus on a research population that is comparable to the European population in terms of ethnicity. The review’s exclusion criteria were (a) studies concerning adopted children or children with intellectual disabilities; (b) studies related to crisis placements, secure residential care, and inpatient psychiatric care; (c) and graduate-level theses or dissertations. No differences were made between articles about kinship foster care (i.e. care by relatives) and non-kinship foster care, due to the ambiguity of evidence in relation to the superior performance of either form of care (Wilson et al. 2004).

We undertook systematic searches with a combination of search terms in the following electronic databases: CINAHL, ERIC, PsychInfo, and MEDLINE. Due to the heterogeneity of the terminology in youth care studies, we used a broad scope of search terms to achieve sufficient coverage of the available literature. Such an approach is common when scoping reviews are conducted (Arksey and O’Malley 2005). First, to define the relevant case characteristics, we used the terms typolog*, epidemolog*, prevalence, profile, baseline, characteristic, discriminat*, variable, cue, differ*, similar*, and compar*. Second, to define the research population we used child*, infant, boy, girl, juvenile, kid, youth, and toddler. Finally, to define settings for out-of-home care we used residential, institutional, foster, out-of-home, group home, shelter care, group care, teaching family homes, family home, family-style group care, teaching family model, and family type home. Thereafter, the results were refined to focus specifically on studies that considered school-aged children (i.e. 6–12 years old) and used the following types of methodology: systematic review, meta-analysis, literature review, prospective study, follow-up study, and longitudinal study. Additional articles were obtained using the snowball method, in which we followed references of interest from relevant handbooks, key journals, and certain articles. Similarly, we hand-searched the sites of relevant organizations that work in the field of youth care, such as the Netherlands Youth Institute.

**Fig. 1:**
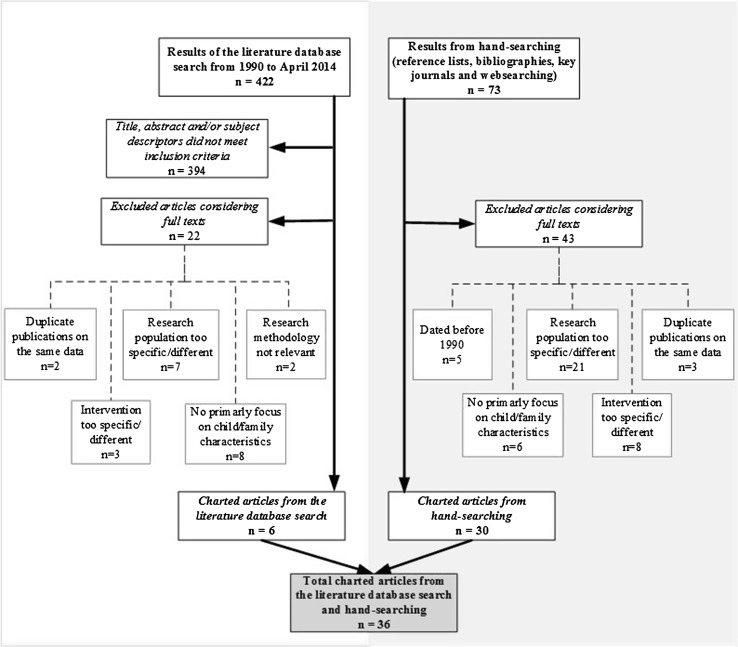
Flowchart showing the results of the search strategy

We determined whether all of the articles identified through the literature search met the inclusion criteria based on their title, abstract, and key words. If they did, their full texts were imported into the “Endnote” bibliographic software package. We then used Microsoft Excel to record several literature data characteristics as the basis for the final selection of articles. The final results of the search strategy, including the specific reasons for article exclusion, are displayed in a flowchart (Fig. 1). Articles that were only used to build the introduction or define specific terms are hereby excluded. In total, 36 articles met all of the inclusion criteria when their full texts were considered. The accompanying Table 1 identifies the considered type(s) of care-modality, sample size, and country of origin considered for each included primary empirical study. Three noteworthy comments can be made with regard to the included articles. First, there was some overlap between the datasets used for analysis in the reports of Strijker et al. (2002, 2005); Hussey (2006); Hussey and Guo (2002); and Tarren-Sweeney (2008, 2013). We nevertheless decided to include all of the articles, due to the different purposes of each study. Second, all of the foster care articles concerned long-term foster care; the sole exception was the article of Lee and Thompson (2008), which specifically related to treatment foster care. Finally, although we used the results of Minnis e tal. (2006) for the description of several characteristics, we excluded their results from our summary table of case characteristics (Table 2). This was because the mostly Caucasian ethnic composition of their population is not comparable with the composition of the European population.

**Table 1:** Summary table of study characteristics of included primary empirical studies (n = 29)

Study (publication year)	Setting(s)^a^	N	Country of origin
Armsden et al. (2000)	FC	362	USA
Barber and Delfabbro (2009)	FC	235	Australia
Bernedo et al. (2014)	FC	104	Spain
Bhatti-Sinclair and Sutcliffe (2012)	OCN	274,203	USA
Esposito et al. (2013)	OCN	2940	Canada
Franzén et al. (2008)	FC, RC	3485^b^	Sweden
Gardeniers and De Vries (2011)	FGC	162	The Netherlands
Holtan et al. (2005)	FC	135	Norway
Hussey (2006)	RC	306	USA
Hussey and Guo (2002)	RC	142	USA
James et al. (2012)	FC, RC	1191	USA
Lee and Thompson (2008)	FC, FGC	828	USA
Minnis et al. (2006)	FC	175	UK
Newton et al. (2000)	FC	514	USA
Scholte (1997)	FC, RC	81	The Netherlands
Scholte and Van der Ploeg (2010)	RC	123	The Netherlands
Strijker and Knorth (2009)	FC	419	The Netherlands
Strijker et al. (2008)	FC	419	The Netherlands
Strijker et al. (2002)	FC	120	The Netherlands
Strijker et al. (2005)	FC	91	The Netherlands
Sullivan (2008)	FC	2996	USA
Tarren-Sweeney (2008)	FC	347	Australia
Tarren-Sweeney (2013)	FC	347	Australia
Van der Steege (2012)	FGC	56	The Netherlands
Vanderfaeillie et al. (2013)	FC	49	Belgium
Vanschoonlandt et al. (2012)	FC	20	Belgium
Vanschoonlandt et al. (2013)	FC	212	Belgium
Yampolskaya et al. (2014)	OCN	33,092	USA
Zima et al. (2000)	FC, RC	330	USA

^a^ *FC* foster care, *FGC* family-style group care, *RC* residential care, *OCN* out-of-home care, not otherwise specified

^b^Only information of the cohort ‘school-aged children (6–12)’ has been used

**Table 2:** Summary table of defining characteristics, arranged by context and setting

	Foster care	Family-style group care	Residential care
*Biological context*			
Male gender/child (%)	38–56	54–62	59–72
Mean age of admission/child (years)	7.5–11.0	10.0–12.0	9.9–13.8
Chronic health problems/child (%)	27–30	7	38
Mean IQ/child^a^	unkn.	unkn.	82.5–90.2
*Individual context*			
Emotional problems/child (%)	14–45	unkn.	39–57
Behavioral problems/child (%)	34–63	40–60	53–62
Attachment problems/child (%)	14–20	50	31–52
School/cognitive problems/child (%)	15–36	30–36	20–55
Use of medication/child (%)	36	unkn.	92
*Family context*			
Divorced/biological parents (%)	84	43	72–80
Deceased/parent (%)	unkn.	27	unkn.
(Physical/emotional) child abuse (%)	5–45	28–52	15–63
(Physical/emotional) child neglect (%)	21–78	39–41	29–69
Child sexual abuse (%)	6–29	17	11–46
Domestic violence (%)	32–41	31	16–18
Parental mental illness (%)	30–61	20–38	41–61
Parental substance abuse (%)	19–34	21	26–49
Parental incarceration (%)	26	16	12
*Care history context*			
Number of previous placements (mean)	1.3–3.4	2.0	4.3–6.6
Admission from birth home (%)	45–56	23	48–52
Child protective service custody (%)	57–59	65–82	66–73
*Social*-*cultural context*			
Peer problems (%)	8	29	46
Caucasian ethnic background (%)	51–58	60–93	49–77
Low income/poverty (%)	81	unkn.	83–95

When percentages or means varied, the range is given

Unkn. = unknown

^a^Total IQ-score

## Results

In this section, the differences and similarities of children at admission to foster care, family-style group care, and residential care that were identified during the literature review are discussed. Additionally, all reported defining characteristics are summarized in Table 2, where they are arranged by both the five contexts of development and the three care modalities.

### Biological Context

Within the biological context, *gender* was frequently mentioned as a defining characteristic. In most studies, girls were more represented in foster care than boys (Armsden et al. 2000; James et al. 2012; Lee and Thompson 2008; Scholte 1997; Strijker et al. 2005, 2008; Van den Bergh and Weterings 2010; Vanderfaeillie et al. 2013; Vanschoonlandt et al. 2013). Some researchers found a slightly higher percentage of boys, up to a maximum of 56 % (Holtan et al. 2005; Minnis et al. 2006; Wilson et al. 2004). Conversely, in family-style group care boys were mostly represented (Gardeniers and De Vries 2011; Lee and Thompson 2008; Van der Steege 2012). Here the reported percentages of boys varied from 54 to 62 %. However, very little evidence was found that the gender differences between foster care and family-style group care are statistically significant. Only Lee and Thompson (2008) reported a significant difference in the number of boys in these two categories. Finally, the vast majority of the children in residential care were boys; the percentages varied from 59 to 72 % (Hussey 2006; Hussey and Guo 2002; James et al. 2012; Lee and Thompson 2008; Scholte 1997; Scholte and Van der Ploeg 2010). Nevertheless, neither James et al. (2012) nor Scholte (1997) found any statistically significant differences between foster and residential care concerning gender differences.

With respect to *age of admission*, children in foster care were on average between 7 and 11 years old (Barber and Delfabbro 2009; Bernedo et al. 2014; James et al. 2012; Minnis et al. 2006; Strijker et al. 2008, 2002). Only Tarren-Sweeney (2013) found an average age of 3.5 years at entry into care, although this presumably concerns the age at *first* placement. In family-style group care, the mean age of admission varied from 10 to 12 years old (Gardeniers and De Vries 2011; Van der Steege 2012). According to Lee and Thompson (2008), children in family-style group care were significantly older than children in foster care when placed out-of-home. However, they only included children aged 8 years and older in their research population, which might have increased the reported mean age of admission. Lastly, the average age of admission for residentially placed children appear to be the highest of the three settings. The reported mean ages varied from 10 to 14 years (Hussey 2006; James et al. 2012; Scholte 1997; Scholte and Van der Ploeg 2010). In comparison with foster children, residentially placed children were reported to be significantly older at admission (James et al. 2012; Scholte 1997). Curtis et al. (2001) made the same conclusion based on their literature review. Only two studies specifically reported age at the time of *first* placement into out-of-home care: Yampolskaya et al. (2014) found an average age of 6.4 years (SD = 5.4), while Hussey and Guo (2002) reported an average of 4.9 (specifically for residentially placed children). It should be noted that the ambiguity in reported figures is presumably due to differences in research methodology between the included studies.

A third defining characteristic of children in care was their *physical health.* Yampolskaya et al. (2014) demonstrated that six percent of the children had physical health problems. However, James et al. (2012) reported substantially more chronic health problems for children in both foster and residential care: they found that approximately one-third of the children have these problems. Likewise, Tarren-Sweeney (2008) indicated physical health problems in 30 % of the foster children. The comparability of the findings related to physical health problems is limited by the heterogeneity of these problems’ definition. Tarren-Sweeney (2008) for example referred to specific physical health problems such as epilepsy and motor neurological conditions, whereas both James et al. (2012) and Yampolskaya et al. (2014) used a broader definition like “the presence of any serious chronic physical health conditions that adversely impact the child’s daily functioning” (Yampolskaya et al. 2014, p. 196).

Lastly, some studies reported the *average IQ* of children in care. A meta-analysis of IQ delays in orphanages by Van IJzendoorn (2008) showed a mean IQ of 84.4 (SD = 16.8), which can be classified as “below average” intellectual functioning. Hussey and Guo (2002) also found a mean IQ of this order for residentially placed children (M = 82.5, SD = 17.4). On the other hand, a longitudinal survey of residentially placed children by Scholte and Van der Ploeg (2010) showed a mean IQ of 90.2, which reflects lower levels of “average intelligence.” Unfortunately, no study was found reporting the mean IQ of foster children and children placed in family-style group care. De Swart et al. (2012) confirmed in their meta-analysis, that even to date remarkable few studies include IQ as moderator, whilst literature data have shown that this factor partly affects the child’s cognitive abilities and learning style. However, a retrospective study by Tarren-Sweeney (2008) concluded that nearly 23 % of foster children had an intellectual disability. In general, available data indicate that a lower IQ is associated with higher levels of psychopathology (Hussey and Guo 2002; Tarren-Sweeney 2008).

### Individual Context

According to Bhatti-Sinclair and Sutcliffe (2012), risk factors within the individual context are the main reason for out-of-home placement. In the literature, a frequently mentioned risk factor was the presence of *emotional problems.* A recent study of Yampolskaya et al. (2014) found that more than half (53 %) of the children in care had such problems. With regard to foster care, the reported percentage of foster children with emotional problems varied from 14 to 45 %, mostly as measured with the Child Behavior Checklist (CBCL) (Armsden et al. 2000; Bernedo et al. 2014; James et al. 2012; Minnis et al. 2006; Scholte 1997; Sullivan 2008; Tarren-Sweeney 2013; Vanderfaeillie et al. 2013). Within residential care, this prevalence rate varied from 39 to 57 % (James et al. 2012; Scholte 1997; Scholte and Van der Ploeg 2010). No information was found regarding emotional problems in children placed in family-style group care. When comparing the number of children with emotional problems in foster and residential care, James et al. (2012) did not find any statistically significant differences. However, Scholte (1997) demonstrated that residentially placed children showed emotional problems significantly more often than foster children.

Considering *behavior problems,* the number of foster children with a score in the (borderline) clinical range on the externalizing problems scale of the CBCL covered a broad area, varying from 34 to 63 % (Armsden et al. 2000; Bernedo et al. 2014; James et al. 2012; Minnis et al. 2006; Tarren-Sweeney 2013; Vanderfaeillie et al. 2013; Vanschoonlandt et al. 2013). At least one-third of foster children had these problems. In contrast, Scholte (1997) reported much lower scores on the different subscales belonging to the externalizing problems scale, varying from 10 to 15 %. This difference is probably due to the dating of the research. Last decades, more children with severe psychosocial problems presumably have been admitted to foster care instead of being placed in more restricted types of care [in accordance with the UN guidelines (2009, December 18)]. In family-style group homes, 40–60 % of the children showed behavior problems, especially hyperactive and impulsive or defiant and antisocial behavior (Van der Steege 2012). Lee and Thompson (2008) found that children in family-style group homes had (with statistical significance) more behavior problems than those placed in treatment foster care. Finally, behavior problems were reported in more than half of the children at admission to residential care (James et al. 2012; Scholte 1997; Scholte and Van der Ploeg 2010). The same studies also reported that residentially placed children showed (with statistical significance) more behavior problems in comparison with foster children. As claimed by Esposito et al. (2013), the degree of behavior problems increases the risk of an out-of-home placement, in particular for older children.

The behavior problems seem in part to be related to *attachment problems* (Newton et al. 2000; Vanschoonlandt et al. 2012). Therefore, the quality of the attachment development of children in care is a third relevant factor within the individual context. A recent review of Pritchett et al. (2013) concluded that the severeness of attachment problems was related to negative placement outcomes. Nevertheless, little detailed information was found concerning the prevalence of the attachment problems of children placed out-of-home. The definition of attachment problems also appeared to be very heterogeneous. Concerning foster care, Tarren-Sweeney (2013) found symptoms in 20 % of the foster children that specifically related to complex attachment problems that were not reducible to other psychiatric disorders. Strijker et al. (2008) reported a slightly lower percentage of 14 %, but they only included foster children with an actual Diagnostic Manual of Mental Disorder classification for reactive attachment disorder. In family-style group care, attachment problems were reported in 50 % of the children (Van der Steege 2012). Finally, Scholte and Van der Ploeg (2010) found signs of social and emotional detachment in 31 % of the residentially placed children. In this study, the Social Emotional Detachment Questionnaire (in Dutch called VFO) was used (Scholte and Van der Ploeg 2007). They have similarly inventoried the rate of children with insecure attachment patterns based on the children’s case files and found a percentage of 52 % (Scholte and Van der Ploeg 2010). Generally speaking, on average one-third of the children in care have attachment problems. This was also confirmed in a meta-analysis by Van IJzendoorn et al. (1999), who demonstrated that 38 % of the children (aged 0–4 years) in “normal” middle class, nonclinical groups in North America showed insecure attachment patterns.

A fourth relevant factor was the *cognitive development* and related *school performance*. As noted previously, both aspects are affected by the child’s intelligence (De Swart et al. 2012). Problems in cognitive development and poor school performance seem to be the least common in foster care; at most one-third of the foster children had poor academic performance (Bernedo et al. 2014; James et al. 2012; Minnis et al. 2006; Scholte 1997; Tarren-Sweeney 2008). Likewise, according to Van der Steege (2012) found that approximately one-third of the children in family-style group care demonstrated cognitive problems such as social skills problems and attention problems. With regard to residential care, the reported percentages of children with cognitive problems showed more variability. One-fifth to one-half of the children appeared to have school-related problems, such as poor school motivation or delays in language, cognition, or adaptive behavior (James et al. 2012; Scholte 1997; Scholte and Van der Ploeg 2010). Zima et al. (2000) found a relationship between caregiver scores in the clinical range on the CBCL and a history of suspension or expulsion. In total, they reported that 14 % of the children in care experienced at least one suspension or expulsion (Zima et al. 2000). These researchers also reported that 23 % of the children in care had reading and math skill delays and that 13 % repeated at least one grade (Zima et al. 2000). Unfortunately, no distinction was made between foster and residentially placed children. James et al. (2012) did not find any significant differences in cognitive development and school performance when comparing residentially placed and foster children. In contrast, Scholte (1997) found significantly more school-related problems in residentially placed children than in foster children. Because different aspects of cognitive development and school performance were measured in the two studies, their results are not directly comparable. In general, both Pritchett et al. (2013) and De Swart et al. (2012) state that little is known about the school performance, cognitive skills, and IQs of out-of-home placed children in relation to placement outcomes. Furthermore, Pritchett et al. (2013) conclude that the existing literature shows conflicting results concerning whether risk factors in this area enhance the chance of negative placement outcomes.

Finally, a study of Tarren-Sweeney (2008) indicated that 36 % of foster children were prescribed any type of *medication*; most common ones being mood-altering (“psychotropic”) and asthma medications. For children in residential care, Hussey and Guo (2002) reported a very high percentage (92 %) of children using psychotropic medication. No studies related to the use of medication in family-style group care were found.

### Family Context

Numbers concerning *parental divorce* were searched first. The percentage of divorced parents (43 %) in family-style group care reported by Van der Steege (2012) approximated the overall divorce rate in the Netherlands, which is 37 % (Centraal Bureau voor de Statistiek 2013). Moreover, 14 % of the children with divorced parents lived in a stepfamily (Van der Steege 2012). The percentage of divorced parents in both foster and residential care is many times higher. In foster care, Scholte (1997) reported a percentage of 84 %. Similarly, in residential care the percentage of divorced parents was indicated as being between 72 and 80 % (Scholte 1997; Scholte and Van der Ploeg 2010). It should be noted that all of the reported percentages are based on Dutch research populations. Also related to the family composition is the percentage *deceased parents*. Numbers were only found for family-style group care. Van der Steege (2012) reported that 9 % of the mothers and 18 % of the fathers of placed children were deceased.

Next to family composition, the degree of family problems was a relevant defining characteristic in children placed out-of-home. Complex and multiple family problems are a main reason for out-of-home placement of young children (aged 0–9 years) in particular (Esposito et al. 2013; Yampolskaya et al. 2014). A commonly mentioned risk factor in this area was child abuse. Concerning *physical or emotional child abuse*, approximately 5–45 % of foster children have a history of this type of abuse (Bernedo et al. 2014; James et al. 2012; Lee and Thompson 2008; Scholte 1997; Strijker et al. 2008; Tarren-Sweeney 2008). Only Minnis et al. (2006) reported a much higher percentage of emotional child abuse in their Scottish sample, namely 77 %. On the other hand, the reported percentage of 5 % by Vanschoonlandt et al. (2013) was actually very low in comparison to other studies concerning foster care. When distinguishing between physical and emotional child abuse among foster children, physical abuse seems to be less common: up to one-third of them have a history of this type of abuse. Regarding family-style group care, Van der Steege (2012) reported a similar percentage of 28 % of children being physically or emotionally abused. In contrast, Lee and Thompson (2008) stated that 52 % of the children in family-style group care experienced physical or emotional abuse. Lastly, the percentage of residentially placed children with a history of this type of abuse varied from 15 to 63 % (Hussey 2006; Hussey and Guo 2002; James et al. 2012; Lee and Thompson 2008; Scholte and Van der Ploeg 2010). It is noteworthy that the Hussey and Guo’s (2002) reported percentage of 63 % was almost twice as high as other reported percentages for residentially placed children. This is possibly due to the specific research population in that study.

Another common type of child abuse was *physical or emotional neglect.* In short, the literature suggests that at least one-quarter to one-third of out-of-home placed children experience neglect, although the presented percentages differ considerably. For foster children, in general one-half to two-thirds of the children have been neglected within their family of origin (Bernedo et al. 2014; James et al. 2012; Lee and Thompson 2008; Strijker and Knorth 2009; Tarren-Sweeney 2008; Yampolskaya et al. 2014). Only Vanschoonlandt et al. (2013) found a much lower percentage of neglected foster children, namely 21 %. Lee and Thompson (2008) found that foster children had a history of neglect significantly more often than children placed in family-style group care. When it comes to this latter type of care, about 40 % of the children have experienced physical neglect, emotional neglect, or both within their family of origin (Lee and Thompson 2008; Van der Steege 2012). In residential care, findings demonstrated percentages of neglected children that varied from 26 to 69 % (Hussey and Guo 2002; James et al. 2012; Lee and Thompson 2008; Scholte and Van der Ploeg 2010). Barber and Delfabbro (2009) stated that in general terms, child neglect mainly occurs in young children. Both Barber and Delfabbro (2009) and Spinhoven et al. (2010) also found that neglected children have an increased risk of other forms of child abuse. In addition, (emotionally) neglected children are most vulnerable for lifetime mood disorders like anxiety or depression in the future (Spinhoven et al. 2010). It therefore seems very important to be alert for signs of child neglect in the event of family problems.

A third form of child abuse was *child sexual abuse.* In foster care, most studies concluded that about 10 % of foster children have been sexually abused in the past (Bernedo et al. 2014; James et al. 2012; Scholte 1997; Strijker et al. 2008; Tarren-Sweeney 2008). At the same time, Minnis et al. (2006) and Lee and Thompson (2008) respectively found percentages of 28 and 29 % in relation to foster children. As far as children in family-style group care are concerned, very little information was found: only a study of Lee and Thompson (2008) reported a percentage of 17 %. This study additionally showed that foster children had a history of sexual abuse significantly more often than children placed in family-style group care. For residentially placed children, the percentage of those who have experienced child sexual abuse in the past appears to be around 10 % (James et al. 2012; Scholte 1997; Scholte and Van der Ploeg 2010). Remarkably, Hussey (2006) reported that almost half of residentially placed children have been sexually abused, whereby girls were almost one and a half times more at risk (61 %) than boys.

Next to child abuse, *domestic violence* was also a relevant risk factor. In foster and family-style group care, domestic violence occurs within about one-third of the families of origin (Lee and Thompson 2008; Strijker et al. 2008; Tarren-Sweeney 2008; Yampolskaya et al. 2014). Lee and Thompson (2008) even reported percentages of 41 % for foster children and 31 % for children in family-style group care, with statistically significant differences between both percentages. As far as residentially placed children are concerned, only Hussey and colleagues reported domestic violence figures. They concluded that such violence occurs within about one-sixth of the families of origin (Hussey 2006; Hussey and Guo 2002).

Furthermore, the presence of *parental mental illness* could be identified as an important risk factor within the family context. In relation to all three types of care, at least one in three parents show mental illness (Hussey and Guo 2002; Lee and Thompson 2008; Scholte 1997; Scholte and Van der Ploeg 2010; Strijker et al. 2008; Van der Steege 2012). However, Scholte and Van der Ploeg (2010) reported that a much higher percentage (61 %) of the parents (of residentially placed children) showed mental illness, whereby mothers clearly more often had these problems (49 %) than fathers (12 %). Likewise, findings of Minnis et al. (2006) demonstrated that 52 % of the biological mothers (of foster children) showed mental illness. Lee and Thompson (2008) reported that the percentage of children in foster care with mentally ill biological parents (45 %) was significantly higher than for children in family-style group care (20 %). In comparing the percentages of mental illness between parents of children in foster and residential care, Scholte (1997) found no significant differences. It should be noted that because of the differences in severeness and kinds of parental mental illness, comparison between the three types of care is limited. In the same vein, this heterogeneity presumably have caused the broad range in percentages of parental mental illness.

Lastly some literature data considered parental substance abuse and parental incarceration. With reference to *parental substance abuse,* in all three types of care at least one in five parents have alcohol or drug problems (Hussey 2006; Hussey and Guo 2002; Lee and Thompson 2008; Strijker et al. 2008; Yampolskaya et al. 2014). Hussey and Guo (2002) even reported drug abuse in 49 % of the parents of children in residential care. Regarding *parental incarceration*, Hussey and Guo (2002) demonstrated that slightly more than 10 % of the residentially placed children had an incarcerated parent. Lee and Thompson (2008) found a similar percentage (16 %) of incarcerated parents for children in family-style group care and a (statistically significant) higher percentage for foster children (26 %).

### Care History Context

To start with, the *mean number of previous placements* was an important defining characteristic. For the Netherlands, we found no literature related to the mean number of placements or repeated referrals to the three care modalities concerned. A large study of Yampolskaya et al. (2014), however, suggested that almost a quarter of the children in care have already experienced a previous placement, of which 29 % have been admitted at least four times since their first referral to youth care. For foster children, some studies reported a mean of 3.1–3.4 previous placements (Lee and Thompson 2008; Tarren-Sweeney 2013). Other studies related to foster care reported a lower mean of previous placements that lied between 1.3 and 1.8 (James et al. 2012; Strijker et al. 2008). Concerning children in family-style group care, Lee and Thompson (2008) concluded that these children have experienced significantly fewer previous placements than foster children, specifically 2.0 placements. Finally, previous placements in residential care appear to be the highest, with an average of at least four (Hussey 2006; Hussey and Guo 2002; James et al. 2012). James et al. (2012) stated that residentially placed children experienced significantly more placements than foster children.

With regard to *admission from birth home,* almost half of the foster children were placed directly from their birth home into foster care during their first out-of-home placement (Barber and Delfabbro 2009; Holtan et al. 2005; Strijker et al. 2008). The former residences of the other half of the foster children in these studies were not clearly reported. Concerning children placed in family-style group care, findings of Gardeniers and De Vries (2011) demonstrated that 23 % of these children entered from their birth home and that approximately the same percentage (22 %) entered from foster care. Most children (48 %) were placed into family-style group care from residential care (Gardeniers and De Vries 2011). Lastly, about half of the children entered residential care from their birth home (Scholte 1997; Scholte and Van der Ploeg 2010), although it could not be determined from the study whether or not this represented a first out-of-home placement. Next to admission from birth home, Scholte (1997) reported that 20 % of the residentially placed children came from a foster family setting while 28 % came from another residential institution.

A final defining characteristic was the percentage of children in *child protective service custody.* When a child is at risk for abuse or neglect or has suffered serious physical or emotional damage, the child can be removed from the custody of his or her parents or guardians by a governmental agency (Arizona Office of the Auditor General 2008). In foster care, the number of children in child protective service custody appears to be the lowest; the reported percentages varied from 57 to 59 % (Strijker et al. 2002; Van den Bergh and Weterings 2010; Vanschoonlandt et al. 2013). A distinction can be made between family supervision and a suspension of parental rights over the child. In the case of suspension, the child is placed under the permanent legal guardianship of the government, and the caseworker has rights and responsibility for the care, custody, and control of the child (DPHHS Human Resources Division 2010). When distinguishing between the two types of custody, Strijker et al. (2002) reported that 19 % of foster children were under family supervision while 13 % were under permanent legal guardianship. In family-home care, at least two-thirds of the children were in child protective service custody, mostly under family supervision (Gardeniers and De Vries 2011; Lee and Thompson 2008; Van der Steege 2012). Finally, approximately 75 % of the children in residential care were in child protective service custody (Hussey 2006; Lee and Thompson 2008; Scholte and Van der Ploeg 2010). Similarly, a review of Frensch and Cameron (2002) also concluded that residentially placed children were mostly under child protective service custody.

### Social-Cultural Context

A first important factor in the social-cultural context was *peer relations.* Results of Scholte (1997) showed that 8 % of foster children experienced problems in this area. He also concluded that such problems were less likely to occur in foster care than in residential care, where a percentage of 46 % was found (Scholte 1997). Minnis et al. (2006) reported in contrast a much higher percentage of 63 % foster children with peer problems in their Scottish sample, based on the Strengths and Difficulties Questionnaire. As far as children in family-style group care are concerned, Van der Steege (2012) reported that 29 % of the children had peer problems.

*Ethnic background* was also a factor that was mentioned often. In general, about half of the children in care have a Caucasian ethnic background (Armsden et al. 2000; Yampolskaya et al. 2014). Nevertheless, the figures concerning ethnic background are hardly comparable due to both the heterogeneity of the defined ethnic groups and the diversity within those groups (Bhopal and Donaldson 1998). For example, “White” or “Caucasian” is often used in American literature; the relevant directive from the U.S. Office of Management and Budget includes people from Europe, North Africa, and the Middle East in the definition of this term (Bhopal and Donaldson 1998). In contrast, the governmental body of Statistics Netherlands considers people from both North Africa and the Middle East to be “non-Western” category (Centraal Bureau voor de Statistiek 2000). This non-Western category also includes people from Africa, Latin America, and Asia. Therefore, the percentages related to ethnic background in our scoping review should be considered as indicative. Several studies reported that more than half of the American children in foster care had a Caucasian ethnic background (James et al. 2012; Lee and Thompson 2008). In contrast, Minnis et al. (2006) reported that 99 % of foster children had a Caucasian ethnic background, but this percentage relates to a Scottish sample and thus is not directly comparable with American foster children. With respect to residentially placed American children, almost half had a Caucasian ethnic background (Hussey 2006; James et al. 2012). In the Netherlands, Scholte and Van der Ploeg (2010) reported a slightly higher percentage of 77 % for residentially placed children. Lastly, a Caucasian ethnic background mostly occurred in family-style group care both in the United States and the Netherlands (Gardeniers and De Vries 2011; Lee and Thompson 2008; Van der Steege 2012). On the other hand, Lee and Thompson (2008) found no statistically significant differences in ethnicity between foster children and children in family-style group care.

A final factor within this context was *social*-*economic status*. James et al. (2012) reported that over 80 % of the children in foster care lived in poverty, based on the number of children with insurance through Medicaid (which is an American social health care program for families and individuals with low income and limited resources). Likewise, more than 80 % of the children in residential care had a low social-economic status (Hussey 2006; James et al. 2012). In a Swedish sample, Franzén et al. (2008) reported lower percentages for out-of-home placed children who are of primary school age. Over 12 % of the mothers were at or below the poverty line. We found no results relating to the social-economic status of children in family-style group care. Overall, both Esposito et al. (2013) and Franzén et al. (2008) concluded that adverse social-economic factors put young children at risk for out-of-home placement.

## Discussion

In general, family-based settings such as foster or family-home care are considered to be the preferred type of care when out-of-home placement is required (Courtney 1998; Doran and Berliner 2001; United Nations 1989). At the same time, the reviewed literature showed that at least one-third of the children placed in family-based settings experience serious placement disruptions (e.g. Scholte 1997; Van den Bergh and Weterings 2010). Several researchers therefore suggest that residential care could sometimes be in the best interests of the child (e.g. Butler and McPherson 2007; De Swart et al. 2012). This suggestion results in the challenge of determining when residential care must be preferred (Frensch and Cameron 2002). However, to date both evidence-based guidelines and assessment tools to make informed decisions for a specific type of out-of-home care are lacking (Barth 2002; Frensch and Cameron 2002; Huefner et al. 2010). To develop such a scientifically supported instrument, insight is needed into the populations referred to the three main care modalities (Barth 2002; Frensch and Cameron 2002). The primary objective of this review was hence to determine similarities and differences in characteristics at admission of school-aged children who were placed in foster care, family-style group care, and residential care.

Notwithstanding the large variation in reported figures, available data indicated the following similarities and differences in case characteristics. In relation to *similarities*, the literature data showed that the majority of normally intelligent children in all three care modalities suffer from severe problems in the individual, family, or social context. Second, several research gaps were found concerning case characteristics at admission to all three types of care. As regards to the individual context, for example, remarkably little is known about intelligence and related cognitive development. The prevalence of attachment problems also remains largely unknown. However, both risk factors appear to relate to placement outcomes (e.g. Pritchett et al. 2013; Tarren-Sweeney 2008). In the family context, figures on domestic violence and sexual abuse were ambiguous or missing in particular. A final research gap in all three care modalities concern care history (such as age at admission and length of stay in care), which was also identified by De Swart et al. (2012). Nevertheless, care history is strongly associated with negative placement outcomes (e.g. Jones et al. 2011; Oosterman et al. 2007).

Meanwhile, available data also revealed various *differences* among children in the three care modalities. Concerning the severity of child and family difficulties at admission, all appear to be most severe in residential care, with the exception of specific parental problems (such as parental mental illness, addiction, and incarceration). In addition, residentially placed children experience the highest number of previous placements, which seems to reflect the tendency to view residential care as the treatment of “last resort” (Barth 2002; Huefner et al. 2010; Nijhof et al. 2014). Our presumption that attachment problems mostly occur in residential care cannot be confirmed, due to an insufficiency of prevalence data regarding the quality of attachment development. In contrast to residential care, problematic family circumstances (and not the individual problems of children) appear to be the main reason for placement in foster care. The high percentages of parents with individual problems such as addiction and mental illness suggest in particular that these problems temporarily preclude parents from offering their children a healthy upbringing. Finally, findings indeed seem to indicate that family-style group care can be considered an intermediate type of care between foster care and residential care, as noted previously (Barth 2002; Huefner et al. 2010; Rouvoet 2009). Most of the reported percentages concerning child and family difficulties at admission of children in family-style group care were between the percentages reported for foster and residentially placed children. In addition, children mostly appeared to enter family-style group care from either foster or residential care.

In summarizing the findings, an initial tentative profile has emerged. Normally intelligent *foster children* could be characterized as young school-aged children whose most notable individual problems include chronic health problems as well as behavioral problems. They usually come from broken, poor families that frequently have histories of neglect and domestic violence. Many parents appear to suffer from mental illness, addiction problems, or both, and one of them would commonly be incarcerated. For *children in family*-*style group care* with average intelligence, the most common finding was that data concerning their individual problems were insufficient. However, the few studies available suggest that attachment and behavioral problems occur particularly frequently and that the children would mostly have a Caucasian ethnic background. With regard to family issues, many children appear to suffer from physical or emotional abuse and are mainly under civil law family supervision. Children placed in family-style group care usually come from another type of care. Finally, *residentially placed children* may be characterized as older school-aged male children with lower than average IQs. Many of them seem to suffer from chronic health problems and the reported figures indicate that many of them are on prescribed medication. Difficulties in peer relations and cognitive problems appear to be the most notable characteristics of residentially placed children, who also seem to frequently display severe emotional and behavioral problems. The extent to which these social-emotional problems relate to attachment problems remains unknown. Furthermore, residentially placed children tend to come from broken, poor families that chiefly have histories of child abuse, neglect, and sexual abuse. Many parents in these families seem to suffer from mental illness and addiction. Literature data suggest that these children are usually under permanent legal guardianship and have experienced an average of at least four placements before they enter residential care.

The results of this review support arguments for the development of an evidence-based assessment tool to make well-informed referral decisions when 24-h out-of-home placement is needed. However, future (longitudinal) research is required to relate intake characteristics to both short- and long-term placement outcomes (Curtis et al. 2001). Other determining factors for out-of-home care should also be considered when developing such an assessment tool, including living group climate (Strijbosch et al. 2015) and the professionalism of youth care workers (De Swart et al. 2012). The hope is that this all will eventually result in optimizing the effectiveness of provided care, given each child’s unique situation.

### Limitations

Some limitations should be noted regarding this scoping review. The first relates to the broad search approach that was used (and is characteristic of a scoping review). In this approach, a study’s substantive relevance is considered to be more important than the methodology used within it (Arksey and O’Malley 2005). However, we still considered this technique to be the most appropriate for answering our research question. The second limitation concerns the considerable variance in the figures reported on the individual and contextual characteristics of children in care, due to the heterogeneity in research methodology, populations, or intervention characteristics of the reviewed studies. For example, the “residential treatment” category in research data includes many definitions, ranging from small-scale community-based settings for 8–10 children to major institutes that are isolated from community life (Curtis et al. 2001; Frensch and Cameron 2002; Huefner et al. 2010). The same is applicable for the terms and definitions used in literature data for foster care (Curtis et al. 2001; Franzén et al. 2008) and family-style group care (Frensch and Cameron 2002; Harder et al. 2013). Third, to deal with the heterogeneity of the terminology utilized in the literature for the three main types of out-of-home care, our search strategy utilized numerous common keywords for every type of care. However, we may have missed particular keywords. Fourth, placement decisions are often dependent on policy of local child care systems or child welfare workers placement preferences (Barth 2002; Bhatti-Sinclair and Sutcliffe 2012; Curtis et al. 2001; Frensch and Cameron 2002; Huefner et al. 2010; James et al. 2004), resource availability (Broeders et al. 2015; Frensch and Cameron 2002; Huefner et al. 2010), and the child’s ethnicity (Becker et al. 2007; Fernandez 1999). This phenomenon has presumably caused large variance in population characteristics and thus limited the generalizability of research findings. Moreover, it also confirms the need for an evidence-based assessment tool for making well-informed referral decisions. Lastly, no uniform definition is available for some constructs (such as ethnic background and attachment), which complicates comparisons between relevant percentages. Such situations were explicitly indicated in the result section.
